# Minimally-invasive mitral valve repair of symmetric and asymmetric Barlow´s disease

**DOI:** 10.1007/s00392-021-01844-9

**Published:** 2021-04-01

**Authors:** Gloria Faerber, Sophie Tkebuchava, Mahmoud Diab, Christian Schulze, Michael Bauer, Torsten Doenst

**Affiliations:** 1 Department of Cardiothoracic Surgery, Jena University Hospital, Friedrich-Schiller-University Jena, Am Klinikum 1, 07747 Jena, Germany; 2 Division of Cardiology, Pneumology, and Intensive Medical Care, Department of Internal Medicine I, Jena University Hospital, Friedrich-Schiller-University Jena, Jena, Germany; 3 Department of Anesthesiology and Intensive Care Medicine, Jena University Hospital, Friedrich-Schiller-University Jena, Jena, Germany

**Keywords:** Mitral valve, Minimally-invasive, Barlow´s disease

## Abstract

**Objectives:**

Barlow´s disease represents a wide spectrum of mitral valve pathologies associated with regurgitation (MR), excess leaflet tissue, and prolapse. Repair strategies range from complex repairs with annuloplasty plus neochords through resection to annuloplasty-only. The latter requires symmetric prolapse patterns and central regurgitant jets. We aimed to assess repair success and durability, survival, and intraoperative outcomes with symmetric and asymmetric Barlow’s disease.

**Methods:**

Between 09/10 and 03/20, 103 patients (of 1939 with mitral valve surgery) presented with Barlow´s disease. All received surgery through mini-thoracotomy with annuloplasty plus neochords (*n* = 71) or annuloplasty-only (*n* = 31). One valve was replaced for endocarditis (repair rate: 99%).

**Results:**

Annuloplasty-only patients were older (64 ± 16 vs. 55 ± 11 years, *p* = 0.008) and presented with higher risk (EuroSCORE II: 4.2 ± 4.9 vs. 1.6 ± 1.7, *p* = 0.007). Annuloplasty-only patients had shorter cross-clamp times (53 ± 18 min vs. 76 ± 23 min, *p* < 0.001) and received more tricuspid annuloplasty (15.5% vs. 48.4%, *p* < 0.001). Operating times were similar (170 ± 41 min vs. 164 ± 35, *p* = 0.455). In three patients, annuloplasty-only caused intraoperative systolic anterior motion (SAM), which was fully resolved by neochords to the posterior leaflet. There were no conversions to sternotomy or deaths at 30-days. Three patients required reoperation for recurrent MR (at 25 days, 2.8 and 7.8 years). At the latest follow-up, there was no MR in 81.4%, mild in 14.7%, and moderate in 2.9%. Three patients died due to non-cardiac reasons. Surviving patients report the absence of relevant symptoms.

**Conclusions:**

Minimally-invasive Barlow’s repair is safe with good durability. Annuloplasty-only may be a simple solution for complex but symmetric pathologies. However, it may carry an increased risk of intraoperative SAM.

## Introduction

Barlow’s disease has been described as billowing mitral leaflet syndrome with an indicative late systolic murmur and non-ejection systolic click [1]. It represents a severe degenerative form of mitral valve disease, where a plethora of pathologies may affect all structures of the valvular apparatus. From a surgical perspective, Barlow's disease is mainly characterized by a diffuse thickened, excessive valvular tissue [2] with elongated and/or thickened chords, severe annular dilatation, pathologic annular motion [3, 4]. Varying degrees of calcification may be observed [5].

The complexity of Barlow´s disease has resulted in different surgical repair strategies: Carpentier's sliding plasty [2], edge-to-edge techniques [6], neochord- or loop-techniques [7, 8], chordal transfer or shortening [9, 10], leaflet flip techniques [11], and “nonresectional” annuloplasty approaches [12–14]. While there is no consensus among surgeons about the optimal technique, there is an agreement that mitral valve repair for Barlow´s disease poses a challenge that requires special training and broad expertise [4, 6, 8]. Thus, Barlow patients may actually receive valve replacement mainly based on the complexity of the pathology. A clear understanding of the various mechanisms of regurgitation is therefore important to be able to repair the valve and provide the best possible outcome.

**Fig. 1:**
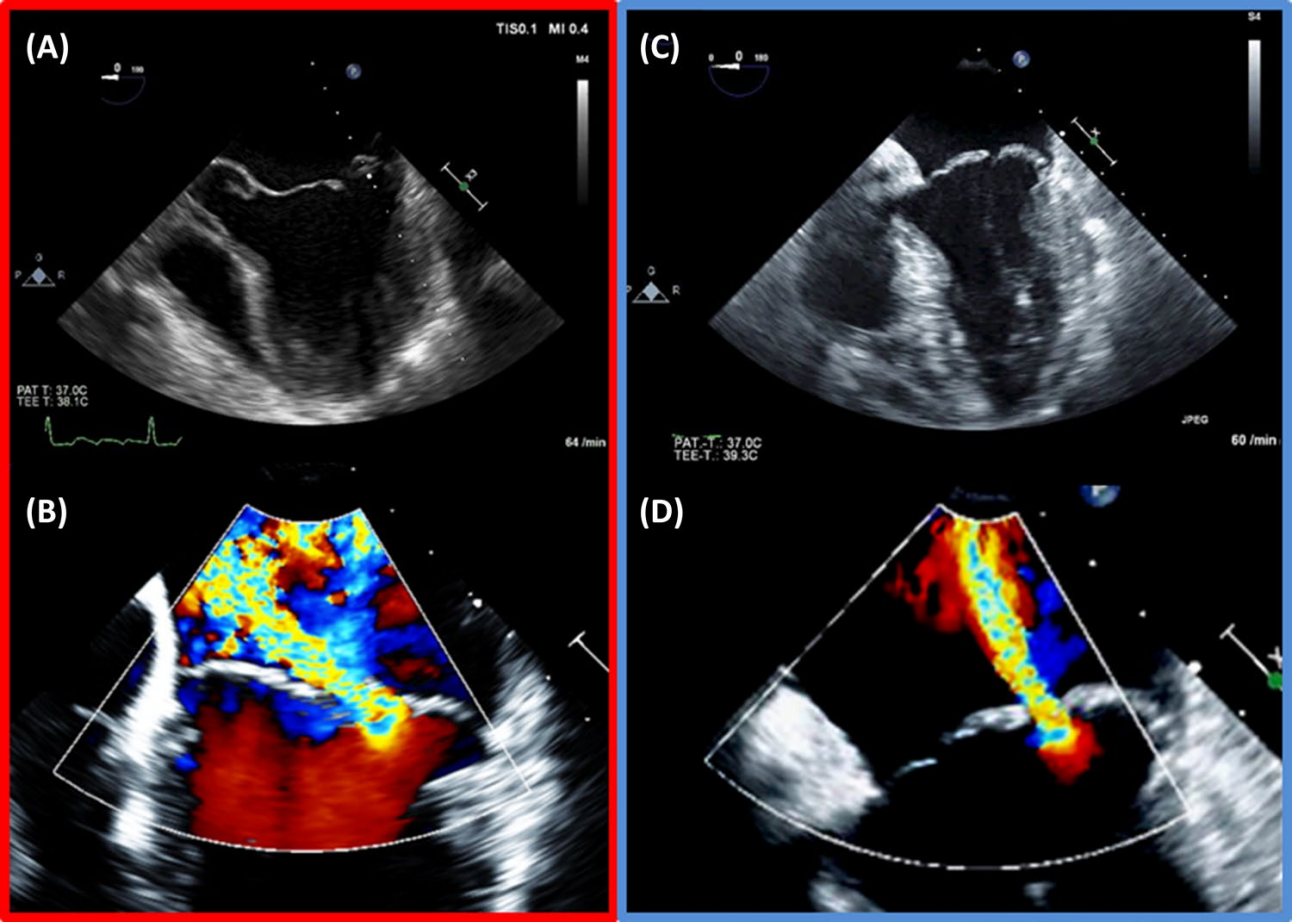
Echocardiographic images of the two different patterns of Barlow´s disease. Asymmetric type with the posterior leaflet predominantly prolapsing (**a**) and anteriorly directed regurgitant jet (**b**). Symmetric type with both leaflets prolapsing to the same height (**c**) and a central regurgitant jet (**d**)

Most surgical Barlow valves present with an “asymmetric pattern” of prolapsing and/or ruptured leaflet segments (Fig. 1a). Echocardiographically, these pathologies are characterized by the presence of eccentric regurgitant jets on color Doppler (Fig. 1b). However, the wide spectrum of pathologies encompasses a subgroup of patients that is characterized by the typical signs of surgical Barlow’s disease but shows a “symmetric pattern” of both leaflets billowing and a central regurgitant jet (Fig. 1c, d). In these cases, we and others [13, 14] have begun to treat those valves with the implantation of annuloplasty only. The purpose of this study was to assess the repair success and durability, survival and intraoperative outcomes with symmetric and asymmetric Barlow’s disease.

## Patients and methods

### Patients

We retrospectively analyzed all patients who underwent mitral valve surgery at our institution since 10/2010. Out of 1939 patients, 103 fulfilled the criteria of surgical Barlow's disease [15]. We define Barlow's disease in our institution as bileaflet prolapse, diffuse excess of billowing leaflet tissue where multiple segments are affected with myxomatous changes [2, 16] and posterior displacement of the posterior annulus towards the left atrial free wall. This definition is consistent with that of others in the literature [2, 15–17], although some investigators have also used the term disjunction for posterior displacement [8, 13, 18] Chords may be elongated, thickened or normal. Severe annular dilatation, pathological annular motion and varying degrees of calcification may be present [3, 5]. One patient was excluded from further analysis due to massive mitral valve endocarditis requiring (uneventful) valve replacement. Analysis of the remaining 102 patients included the success rate of mitral valve repair (primary endpoint) and durability of mitral valve repair as well as survival and intraoperative outcomes (secondary endpoints) with symmetric and asymmetric Barlow’s disease. Patient´s data (demographic, perioperative, and follow-up information) were obtained from charts, medical records, and telephone interviews. Follow-up was routinely performed yearly by mail, which we completed for this analysis by individually contacting and/or seeing each patient. Follow-up had a mean duration of 2.7 years (11 days to 9.2 years) and was 100% complete. It consisted of assessing NYHA class, echocardiography (examinations form external cardiologists were also accepted), re-do surgery for recurrent mitral valve regurgitation (MR) as well as further assessments in the rare case-patients were symptomatic. The severity of mitral valve regurgitation (MR) was graded as none, mild, moderate, and severe according to current guidelines [19]. The local ethics committee approved this retrospective study (reference number: 2020–1712).

### Surgical technique

All patients were operated with a minimally-invasive approach using a right-sided mini-thoracotomy (5 cm) through the 4th intercostal space. Cardiopulmonary bypass (CPB) was established through percutaneous groin cannulation as described by us before [20]. The aorta was cross-clamped with a Chitwood clamp [21] and Bretschneider's cardioplegic solution was used for myocardial protection. The left atrium was entered through Waterston's groove. The general principle of our mitral valve repairs consists of re-suspension of prolapsing or flail segments using neochords (a set of four premeasured Gore-Tex loops are anchored at the papillary muscle and individually sutured to the free leaflet margin for length adjustment or refixation) combined with ring annuloplasty. The Carpentier-Edwards Physio annuloplasty ring (Edwards Lifesciences®) was used in 59 (57.8%), the rigid saddle ring (RSAR, Abbott®) in 41 (40.2%) and the Cosgrove-Edwards annuloplasty system (Edwards Lifesciences®) in 2 (2.0%) patients for annuloplasty. Additional techniques as resection, commissuroplasty or cleft closure were added as judged by the operating surgeon. Annuloplasty only was performed if the preoperative echocardiography showed symmetric billowing of both leaflets combined with a central jet (Fig. 1c, d). Additional procedures such as the closure of an atrial septal defect, patent foramen ovale, cryoablation, closure of the left atrial appendage, myectomy or tricuspid valve repair were performed as required.

### Statistical analysis

Continuous variables are presented as mean ± standard deviation, categorical variables as absolute and relative frequencies. Differences between continuous variables were compared by Student’s t-test or Welch’s t-test. Categorical data were compared using Chi-square, Fisher’s exact or Mann–Whitney-U test. The Kaplan–Meier method was used to analyze time-to-event data on survival, recurrent MR, and mitral valve reoperation as the log-rank and Wilcoxon test were used for group comparisons. The significance level was set at *p *< 0.05. All statistical analyses were performed with SPSS software (IBM SPSS Statistics for Windows, Version 25).

## Results

### Baseline characteristics of the study population

Table 1 shows preoperative and demographic data of the patient population divided according to the mitral valve treatment received. The primary endpoint of repair success was achieved in all 102 patients, which results in a repair rate of 99% considering the one patient that required replacement of massive endocarditis. The mitral valve was repaired by implantation of annuloplasty plus neochords in 71 (70%) patients and by annuloplasty only in 31 (30%) patients. Annuloplasty only patients were older (64 ± 16 vs. 55 ± 11 years, *p* = 0.008) and more often female (45.2% vs. 23.9%; 0.038). Patients of both groups were mainly symptomatic at NYHA class II to III, and represented a low-risk cohort with STS score of 1.1 and EuroSCORE II of 2.4. Annuloplasty only patients presented with more comorbidities and a higher surgical risk profiles (EuroSCORE II: 4.2 ± 4.9 vs. 1.6 ± 1.7, *p* = 0.007 and STS-Score: 1.8 ± 1.7 vs. 0.7 ± 0.5, *p* = 0.612).

**Table 1:** Demographic and baseline characteristics of the patient population divided according to the mitral valve treatment received: annuloplasty plus neochord vs. annuloplasty only

	Annuloplasty plus neochord (*n* = 71)	Annuloplasty only (*n* = 31)	p-value
Age [year]	55 ± 11.0	64 ± 15.5	0.008
Female (%)	17 (23.9%)	14 (45.2%)	0.038
NYHA class	2.3 ± 0.7	2.5 ± 0.7	0.115
I	10 (14.1%)	3 (9.7%)	
II	33 (46.5%)	10 (32.3%)	
III	26 (36.6%)	17 (54.8%)	
IV	2 (2.8%)	1 (3.2%)	
Arterial hypertension	46 (64.8%)	22 (71.0%)	0.543
IDDM	3 (4.2%)	3 (9.7%)	0.365
Pulmonary hypertension	29 (40.8%)	19 (61.3%)	0.057
COPD, treated	4 (5.6%)	3 (9.7%)	0.432
Coronary artery disease	4 (5.6%)	6 (19.4%)	0.063
Atrial fibrillation	17 (23.9%)	17 (54.8%)	0.002
s.p. cardiac surgery	0	1 (3.2%)	0.304
Stroke	3 (4.2%)	5 (16.1%)	0.054
Renal failure	5 (7.0%)	4 (12.9%)	0.449
Dialysis dependent	0	1 (3.2%)	0.304
EuroSCORE II	1.6 ± 1.7	4.2 ± 4.9	0.007
STS-Score	0.7 ± 0.5	1.8 ± 1.8	0.612

Data are given in n (% of total) or mean ± standard deviation. *NYHA* New York Heart Association functional classification, *IDDM* insulin-dependent diabetes mellitus, *EuroSCORE* European System for Cardiac Operative Risk Evaluation, *STS* Society of Thoracic Surgeons

### Perioperative characteristics and in-hospital outcomes

Table 2 shows the intraoperative echocardiographic data of the annuloplasty plus neochord and the annuloplasty only groups. Annuloplasty only patients tended to present with slightly lower ejection fraction, smaller end-diastolic dimensions, and less severe MR under general anesthesia. They also showed more valvular calcification. There was no flail leaflet in the annuloplasty only patients.

**Table 2:** Intraoperative echocardiographic characteristics

	Annuloplasty plus neochord (*n* = 71)	Annuloplasty only (*n* = 31)	p-value
Ejection fraction [%]	63 ± 8.2	59 ± 10.1	0.052
LVEDD [mm]	57.8 ± 8.3	50.8 ± 6.7	0.001
LVESD [mm]	36.5 ± 7.6	32.6 ± 8.1	0.092
LADs [mm]	47.0 ± 8.5	47.7 ± 10.9	0.776
Left atrial volume [ml]	69.7 ± 38.7	63.2 ± 13.2	0.690
MR grade	3.0 ± 0.1	2.9 ± 0.3	0.014
None	0	0	
Mild	0	0	
Moderate	1 (1.4%)	4 (12.9%)	
Severe	71 (98.6%)	27 (87.1%)	
MV annulus [mm]	42.0 ± 5.0	39.6 ± 4.7	0.173
VC [mm]	7.3 ± 1.7	7.1 ± 1.7	0.633
EROA [cm^2^]	0.5 ± 0.2	0.5 ± 0.3	0.817
Regurgitant volume [ml]	66.6 ± 27.4	65.3 ± 16.3	0.906
Calcification	14 (19.7%)	7 (22.6%)	0.742
Flail leaflet	28 (39.4%)	0	< 0.001

Data are given in n (% of total) or mean ± standard deviation.* LVEDD*  left ventricular end-diastolic diameter, *LVEDD*  left ventricular end-systolic diameter, *LADs*  left atrial dimension in systole, *MR*  mitral regurgitation, *MV*  mitral valve, *VC*  vena contracta, *EROA* effective regurgitant orifice area

**Fig. 2:**
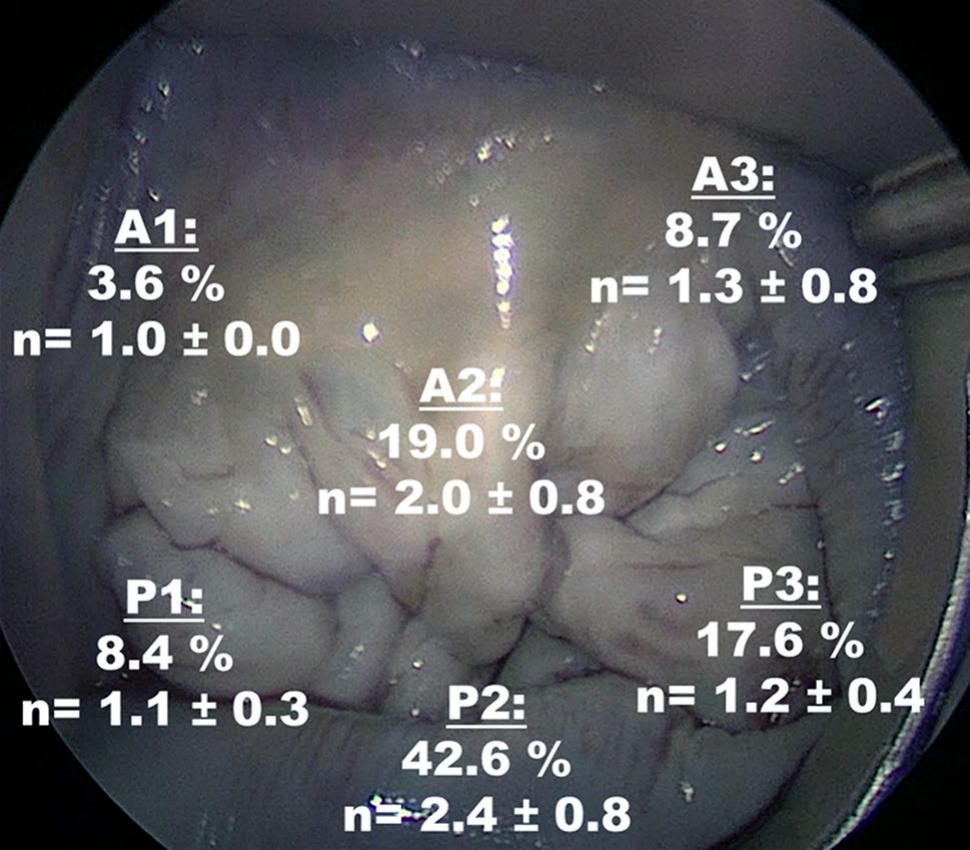
Illustration of the localization and average numbers (*n*, mean ± standard deviation) of neochords used. Neochords were most commonly placed to the P2 (42.6%) followed by A2 (19.0%) and rarely to the A1 segment (3.6%) with an average number of two cords for the P2 / A2 segment and one chord to the remaining segments

Table 3 shows the operative characteristics and in-hospital outcomes. All patients received a minimally-invasive approach. Annuloplasty plus neochord and annuloplasty only patients had similar operating (170 ± 41 min vs. 164 ± 35 min; *p* = 0.455) and cardiopulmonary bypass times (133 ± 35 min vs. 126 ± 30 min; *p* = 0.219). Cross-clamp time was shorter for annuloplasty only (53 ± 18 min vs. 76 ± 23 min, *p* < 0.001). In the annuloplasty plus neochord group, neochords were predominantly placed to the posterior leaflet (34 patients), followed by placement to both (32 patients) and to the anterior leaflet (5 patients). Neochords were most commonly placed to the P2 (42.6%) followed by A2 (19.0%) and rarely to the A1 segment (3.6%) with an average number of two cords for the P2 / A2 segment and one chord to the remaining segments (Fig. 2). Resection at the posterior leaflet was performed in 4, cleft closure in 6 and commissuroplasty in three patients. In the annuloplasty only group, there were no resections; cleft closure was performed in 4 and commissuroplasty in one patient. The mean ring size was 34 mm. In the annuloplasty plus neochord group, rings were larger (35 ± 3.2 mm vs. 33 ± 3.0 mm; *p* = 0.011).

**Table 3:** Operative characteristics and in-hospital outcomes

	Annuloplasty plus neochord (*n* = 71)	Annuloplasty only (*n* = 31)	p-value
Duration of operation [min]	170 ± 41	164 ± 35	0.455
CPB time [min]	133 ± 35	126 ± 30	0.219
Cross-clamp time [min]	76 ± 23	53 ± 18	< 0.001
Neochordae	71 (100%)	–	< 0.001
Posterior leaflet	–	–	
Anterior + posterior leaflet	32 (45.1%)	–	
Anterior leaflet	5 (7.0%)	–	
Tissue resection	4 (5.6%)	–	0.311
Cleft closure	6 (8.5%)	4 (12.9%)	0.487
Commisuroplasty	3 (4.2%)	1 (3.2%)	> 0.999
Mean ring size [mm]	35 ± 3.2	33 ± 3.0	0.011
Concomitant procedures			
ASD/PFO closure	57 (80.3%)	23 (74.2%)	0.492
Cryoablation	15 (21.1%)	11 (35.5%)	0.126
LAA closure	14 (19.7%)	11 (35.5%)	0.089
Myectomy	2 (2.8%)	1 (3.2%)	> 0.999
Tricuspid valve repair	11 (15.5%)	15 (48.4%)	< 0.001
Adverse events			
Rethoracotomy, bleeding	2 (2.8%)	0	> 0.999
New onset of AF	3 (4.2%)	0	0.551
Stroke	1 (1.4%)	0	> 0.999
Wound infection	0	1 (3.2%)	0.304
Renal failure, dialysis	0	1 (3.2%)	0.304

Data are given in n (% of total) or mean ± standard deviation. *CPB* Cardio pulmonary bypass, *LAA*  Left atrial appendage, *ASD*  Atrial septal defect,* PFO*  Patent foramen ovale, *CPR*  Cardiopulmonary resuscitation, *AF*  Atrial fibrillation

In three patients (presented here as part of the annuloplasty plus neochord group), annuloplasty only resulted in intraoperative systolic anterior motion (SAM: 9% of all annuloplasty only patients). The treatment strategy in the first patient was resection and sliding plasty of the posterior leaflet plus neochord and implantation of a larger ring (38 to 40 mm). The second patient received neochords to the anterior and posterior leaflets in addition to the already implanted 38 mm ring. In the third patient, neochords were inserted into the posterior leaflet as an addition to the annuloplasty ring. In all three cases, SAM was fully resolved within the second cross-clamp time without residual MR, good valvular function and normal gradients.

Concomitant procedures were the closure of an atrial septal defect or patent foramen ovale, cryoablation, closure of the left atrial appendage, myectomy or tricuspid valve repair. The annuloplasty only group received more tricuspid valve repairs, which consisted exclusively of band annuloplasty. There were no intraoperative deaths or conversions to sternotomy.

Table 3 also shows postoperative complications. While in the annuloplasty plus neochord group, re-operation for bleeding was necessary in two patients, none of the annuloplasty only patients underwent re-thoracotomy. In both groups, 30-day mortality was 0%. New onset of atrial fibrillation was seen in three annuloplasty plus neochord patients. Stroke occurred in one annuloplasty plus neochord patient with already preoperative recurrent transient ischemic attack in the context of endocarditis. Wound infection was seen in one female annuloplasty-only patient at the mini-thoracotomy site. She additionally suffered from acute kidney injury and temporarily required dialysis.

Discharge echocardiography showed no MR in 88 (86.3%), trace in 11 (10.8%) and mild in 3 (2.9%) patients. None of the patients showed moderate or severe MR at discharge.

### Follow-up and long-term outcome

**Fig. 3:**
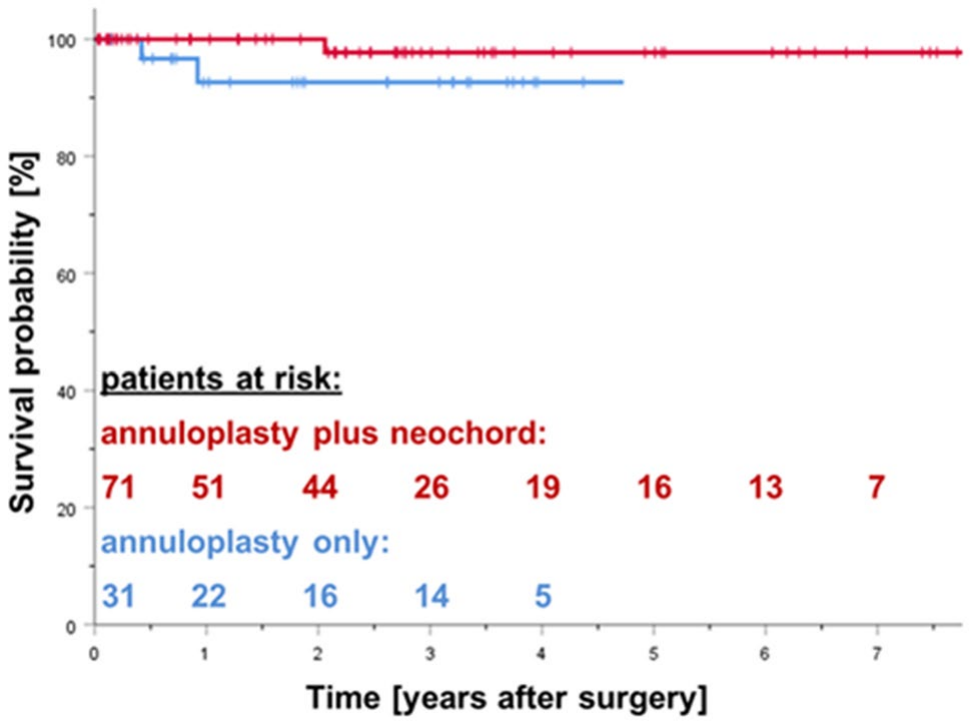
Kaplan–Meier survival estimate after minimally invasive mitral valve repair with annuloplasty plus neochord (red) and annuloplasty only (blue) patients (log Rank 0.183, n.s.). Curves were truncated when the number of patients at risk decreased below *n* = 5

Figure 3 shows Kaplan–Meier survival curves after mitral valve repair. There were three late deaths, due to non-cardiac reasons. In the annuloplasty plus neochord group, one patient died due to cancer on day 753. In the annuloplasty only group, two patients died due to pulmonary disease and pneumonia on day 153 and 335. There was no significant difference in survival between the two groups (Log Rank 0.183).

**Fig. 4:**
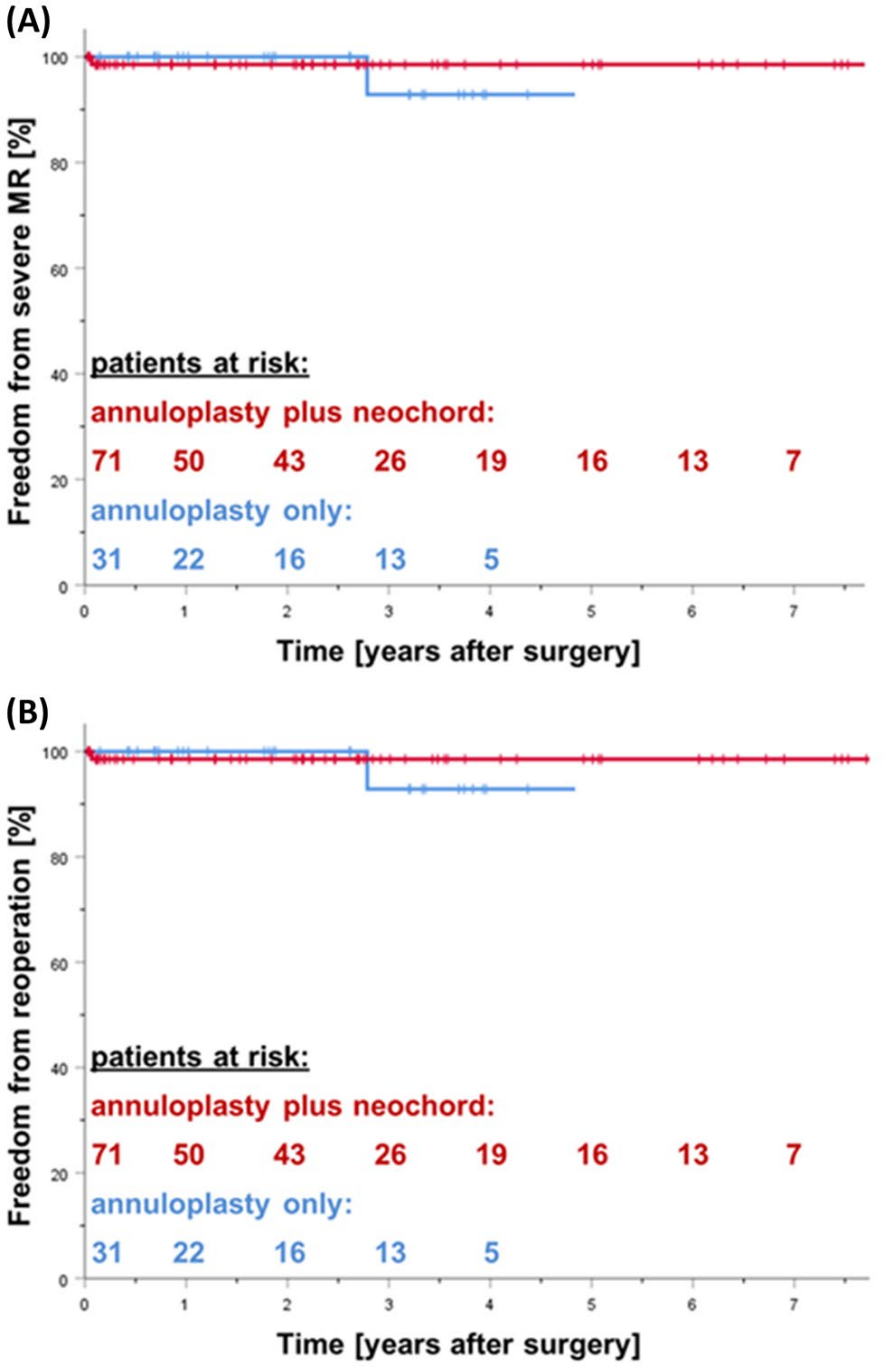
Freedom from severe mitral regurgitation (**a**) for annuloplasty plus neochord (red) and annuloplasty only (blue) patients (log Rank 0.183, n.s.) and Freedom from mitral valve reoperation (**b**) for annuloplasty plus neochord (red) and annuloplasty only (blue) patients (log Rank 0.183, n.s.). Curves were truncated when the number of patients at risk decreased below* n* = 5

Table 4 shows clinical outcome and echocardiographic data during follow-up. At latest follow-up, most patients were asymptomatic in NYHA class I (*n* = 83, 81%). Annuloplasty only patients presented with slightly higher NYHA class (NYHA class 1.4 ± 0.6 vs. 1.1 ± 0.3, *p* = 0.003), who already presented with higher preoperative risk (Table 1). The most recent echocardiographic follow-up showed durable results after valve surgery without signs of SAM, ring dehiscence or rupture of implanted neochords. Almost all patients presented with normal left ventricular function. Freedom from more than moderate MR was similar for both groups (annuloplasty plus neochord: 97% vs. annuloplasty only: 94%; Fig. 4a). Three patients underwent re-operation (Fig. 4b). The mechanism of recurrent MR in two patients (annuloplasty plus neochord group: *n* = 1 on day 25, annuloplasty only group: *n* = 1 after 2.8 years) was a newly ruptured chord at the posterior leaflet. Both were successfully re-repaired minimally-invasively by placing additional neochords to the torn segments. The reason for MR in the third patient (annuloplasty plus neochord group) was progressive degeneration of the anterior leaflet 7.8 years after mitral valve repair. The valve was replaced.

**Table 4:** Outcome and follow-up data

	Annuloplasty plus neochord (*n *= 71)	Annuloplasty only (*n* = 31)	p-value
NYHA class	1.1 ± 0.3	1.4 ± 0.6	0.003
I	58 (81.7%)	20 (64.5%)	
III	11 (15.5%)	9 (29.0%)	
II	2 (2.8%)	2 (6.5%)	
IV	0	0	
Follow-up echo			
MR, grade	0.2 ± 0.5	0.3 ± 0.7	0.834
None	58 (81.7%)	25 (80.6%)	
Mild	11 (15.5%)	4 (12.9%)	
Moderate	2 (2.8%)	1 (3.2%)	
Severe	0	1 (3.2%)	
Ejection fraction [%]	59.9 ± 8.7	59.8 ± 10.0	0.902

Data are given in n (% of total) or mean ± standard deviation. *NYHA*  New York Heart Association, *MR*  mitral regurgitation

## Discussion

We demonstrate in this study that minimally-invasive mitral valve repair for Barlow´s disease is safe and provides good durability. Annuloplasty only maybe a simple solution for complex but symmetric pathologies. However, it seems to carry an increased risk of intraoperative SAM.

Barlow´s disease reflects a heterogeneous complex of pathologies affecting all components of the mitral valve apparatus. Other surgeons have presented very good long-term results with their individual repair strategy [6, 8], suggesting that different solutions are appropriate and possible for successful treatment. Our repair concept consists primarily of re-suspension (i.e., reattaching or shortening) of prolapsing segments with premeasured Gore-Tex loops in combination with ring annuloplasty. With this strategy, our results appear consistent with the favorable outcomes of others. Our repair rate was near 100% which is technically identical to the results of Adams et al. in a mixed cohort of patients with structural MR [15]. We experienced no need for conversion and did not lose a patient in the hospital. Our long term freedom from recurrent MR and reoperation rates range above 90% which is again consistent with the reports of others. [6, 8, 22].

With our specific interest in mitral valve surgery, we noticed a fraction of patients, who presented with a symmetric pattern of Barlow’s disease. Similar to those with asymmetric pattern, we found characteristic signs of Barlow´s disease but without chordal rupture and a central regurgitant jet on echocardiographic examination. For Barlow´s disease, a larger annulus with pathological shape and movement has been described as characteristic to leaflet and chordal pathology [23]. Particularly, pathological annular movement in late systole is considered to lead to complete prolapse of the entire valve with all segments billowing to the same height and symmetric level causing MR [12–14]. In addition, loss of “diastolic mitral valve locking” (defined as sustained diastolic closure of the mitral valve after atrial systole) [24]; decoupling of annular and ventricular contraction [18, 23] and a paradoxical motion of the papillary muscles towards the annular plane in systole (due to exerted traction of the prolapsing leaflets) [12, 25] have been described to aggravate mitral valve prolapse, putting stress on chords and cause MR. All these pathomechanisms share a common feature: They may be addressed at the annular level.

Since ring annuloplasty alone results in significant remodeling of mitral valve function [12–14], we and others [12–14] reasoned that the implantation of an annuloplasty ring only might suffice as a repair technique. Although isolated ring annuloplasty represents a simple technique in terms of its execution, it implies a complexity within the decision-making process especially ring sizing and SAM. We chose our ring sizes in this patient population based on matching the sizer to the surface of the anterior leaflet [15]. The annuloplasty resulted in the relocation of both leaflets and the positioning of the coaptation zone below the annular plane (i.e., leaflet coaptation moved into the ventricle). Thus, we successfully addressed the above-described pathomechanisms.

Nevertheless, isolated ring annuloplasty also increased the risk of SAM. SAM occurred in 3 of 34 patients, where it was absent in the other group. Others report SAM with annuloplasty only strategy in 7/24 (29%) patients [14] and 2/41 (5%) patients [13], thus, supporting our findings. Treatment strategies included medical treatment or surgical revision [13, 14]. We placed Gore-Tex chords to the posterior leaflet in all three cases which shifted the coaptation line posteriorly and fully resolved SAM in all cases. The pathophysiology of SAM is influenced by many different factors and we cannot draw any solid conclusions based on this dataset. However, our experience has guided us to assessing ventricular dimensions, geometry of the outflow tract, and to select larger rings than suggested by our described sizing strategy for symmetric pathologies. This strategy is consistent with that of other experts in the field [14, 26].

The avoidance of resection or re-suspension of diseased leaflet tissue raises the question of durability. We found comparable mid-term outcomes in our annuloplasty only patients compared to our annuloplasty plus neochord patients and to the findings of others [7, 8, 12–14]. However, it may be argued that the one patient requiring reoperation during follow-up for chordal rupture may have been prevented if Gore-Tex loops had been placed. While this argument may sound convincing, the logic is not, because it is not predictable which chord will tear in the future. Even in asymmetric Barlow´s disease, not all segments are resected or re-suspended. The one case in our asymmetric group, where a chord ruptured that was not re-suspended may serve as proof of principle for this statement. It has been previously discussed that causes for recurrent MR after initially successful repair may be related to the surgical technique or to the underlying disease. The latter has been shown to be the most important factor for post-repair failure with the highest recurrence rates for Barlow´s disease (6% per year vs. 2.6% for fibroelastic deficiency) [27–31]. However, our current results suggest lower repair failure rates also for Barlow patients.

Besides the good durability of our repair strategies, another important consideration is the fact that all our Barlow’s´ patients were operated minimally-invasively. There was not a single sternotomy case and also not a single conversion. Even the re-repairs were performed as minimally invasive re-operations. This result underscores conclusions also drawn by others that minimally-invasive access is possible in all patients and does not limit valve repair [6–8]. As already addressed above, even complex cases with severe calcifications or septal hypertrophy and re-operations were treated through mini-thoracotomy. Our surgical approach to the valve does not differ between minimally-invasive and sternotomy access, while sternotomy is only used if there are convincing reasons not to use the minimally-invasive access. The results also show that this complex pathology can be reproducibly repaired with a high likelihood of success without the need for replacement, which would most likely produce an inferior patient prognosis.

## Limitations

Our analysis has several limitations. First, it is a retrospective single-center experience. Second, the documented durability appears promising, although follow-up may not be considered long-term, yet. Nevertheless, there is hardly any dynamic in mitral regurgitation from discharge to the latest follow-up. Third, four patients with symmetric Barlow’s pathology received annuloplasty plus neochords who were characterized by a massive excess of valvular tissue and very large annuli. In these cases, we anticipated a high risk of SAM with the annuloplasty only approach. All of these patients received the largest available annuloplasty ring (40 mm) and additional neochords. We can therefore not determine, based on this retrospective analysis, whether annuloplasty only would have worked in these patients as well. The information, however, does not limit our conclusion that annuloplasty alone is a possible option in selected patients. Finally, the comparison of annuloplasty plus neochord and annuloplasty only patients is limited by the inter-group differences in baseline characteristics. In order to fully address comparability, a prospective randomized approach would be needed. In any case, these data are hypothesis-generating and suggest that in selected symmetric Barlow cases, isolated annuloplasty may give a good outcome with the potential for durable results.

## Conclusion

Minimally-invasive mitral valve repair for Barlow’s disease is safe and provides good durability. Annuloplasty only maybe a simple solution for complex but symmetric pathologies. However, it seems to carry an increased risk of intraoperative SAM.
